# From Point to Filament Defects in Hybrid Nematic Films

**DOI:** 10.1038/s41598-019-50948-w

**Published:** 2019-11-29

**Authors:** Cesare Chiccoli, Paolo Pasini, Claudio Zannoni, Gregor Skačej, Hiroyuki Yoshida, Taiga Hiroshima, Kanta Sunami, Tomohiro Ouchi, Masanori Ozaki

**Affiliations:** 1 INFN Sezione di Bologna, Via Irnerio 46, 40126 Bologna, Italy; 2 Dipartimento di Chimica Industriale Toso Montanari, Università di Bologna and INSTM, Viale Risorgimento 4, I-40136 Bologna, Italy; 3 Faculty of Mathematics and Physics, University of Ljubljana, Jadranska 19, SI-1000 Ljubljana, Slovenia; 4 Division of Electrical, Electronic and Informational Engineering, Osaka University, 2-1 Yamada-oka, Suita-shi, Osaka-fu 565-0871 Japan; 5 JST, PRESTO, 4-1-8 Honcho, Kawaguchi, Saitama 332-0012 Japan

**Keywords:** Condensed-matter physics, Liquid crystals

## Abstract

We have studied nematic hybrid films with homeotropic alignment at the top surface and various controlled degrees of in plane ordering, going from a random degenerate organization to a completely uniform alignment along one direction, at the bottom one. We show, by Monte Carlo (MC) computer simulations and experiments on photopatterned films with the bottom support surface fabricated with in-plane order similar to the simulated ones, that the point defects observed in the case of random planar orientations at the bottom tend to arrange along a filament as the surface ordering is sufficiently increased. MC simulations complement the polarized microscopy texture observations allowing to inspect the 3D structure of the defects and examine the role of elastic constants.

## Introduction

Contrary to what the name would suggest, defects are not necessarily a negative feature of materials. Indeed “defect engineering” is a blooming methodology for fabricating novel complex molecular organizations starting from liquid crystal topological defects^1–15^. In essence, the approach is normally based on the fact that defects correspond to high free energy regions of the system, which makes them prone to be attacked by suitable reagents or, in turn, capable of attracting colloidal or nano-particles in an effort to reduce the local free energy excess.

The methodology has been employed, e.g. to prepare ordered arrays of gold or other metal nanoparticles with the goal to prepare self-assembling metamaterials^4^ and even active bacterial system^16^. Defects in liquid crystals correspond to well defined categories endowed with a certain topological charge *s*^17,18^, and their positions and nature can, to some extent, be controlled from outside, employing suitable boundary conditions and external fields. For instance in a thin film of nematic spread on a spherical colloidal particle, where the total topological charge should be 2, four *s* = 1/2 defects placed in a tetrahedral arrangement appear, making the particle a kind of tetravalent “colloidal atom”^2^. This valence can, however, be changed by the application of suitable multipolar fields^3^ while the defect location can be changed, e.g. varying the shape of the colloidal core from spherical to ellipsoidal^19^. In thin nematic films, sandwiched between two flat surfaces with a null total topological charge (*s* = 0), the occurrence and nature of defects can also be controlled by imposing, apart from external fields, various types of boundary conditions with the help of appropriate surface anchoring agents. Thus, defects should be absent in a flat cell with a uniform alignment either parallel to a certain direction of the surface, **r**, or tilted with respect to it, realizing a monodomain. More generally, when the film is so thin that the director can be considered to live in two dimensions (2D), homotopy theory shows that there can be in principle infinitely many types of disclinations in director orientations classified by their semi-integer or integer topological charge *s*^20–22^. These various defects have of course different free energy and in practice only the ones with smallest *s* are normally observed. The situation is different in the bulk, where only half integer defects are allowed, while integer ones can be eliminated and relax out, e.g. by the escape in the third dimension mechanism^23^.

In the present work, we are interested in nematic films with hybrid boundary conditions, where the liquid crystal has planar degenerate anchoring at the solid support surface and homeotropic alignment at the top interface, defects will appear when the film thickness *h* is much smaller than the lateral size *L*^24^. This kind of stable defects for hybrid systems has been investigated experimentally and on the basis of continuum theory (see for example^25^) as well as by lattice spin simulations^24^. In particular, we have shown in the past that Monte Carlo computer simulations of lattice spin models with nearest neighbor interactions like the Lebwohl-Lasher^26,27^ one, where only particle orientations are considered, can reproduce the appearance of stable point defects in hybrid nematic films^24^. In practice these contrasting boundary conditions are implemented in the simulations with two additional, static, layers of spins where those at the bottom (*z* = 0) have random fixed orientations in the horizontal (*x*, *y*) plane, while those of the top (*z* = *h*) layer are fixed along the surface normal^24^. It has also been reported that disclination lines can be formed in a hybrid nematic cell where the two facing flat surfaces enclosing the nematic are respectively treated to induce an orientation perpendicular to the surface at the top (*z* = *h*), as in the previous case, and an homogeneous, uniform, in plane alignment along *x* or *y* at *z* = 0^28^. However, the surface order needs not to be complete and, more generally, the extent of ordering at the surface can be quantified introducing a two dimensional surface order parameter 〈*T*_2_〉^29^,

1 $$\langle {T}_{2}\rangle =\langle \,\cos \,(2\alpha )\rangle ,$$

where *α* is the orientation of a molecule belonging to the surface plane with respect to the global easy axis of the surface. The two limiting situations just described correspond to 〈*T*_2_〉 = 0 (planar degenerate) or 1 (perfect alignment along one direction in the plane). The effect on the molecular organization inside a nematic film in this intermediate situation with only partial surface order is not obvious, particularly for what concerns the type of defects formed. As an example, it is not known, as far as we are aware, if there is some critical surface order that enforces the transition from points to lines or walls, everything else being the same, or actually which type of defects occurs.

Clarifying the way in which specific defect organizations can be created is clearly important, also in view of the aforementioned applications, and in the present paper we wish to study how the point defects transform by changing the bottom surface of the hybrid film from a random planar molecular orientations to a fully ordered one by gradually increasing the percentage of ordering. We tackled the problem both by Monte Carlo computer simulations and real experiments. Simulations allow to precisely define the surface order and to provide input for preparing similarly ordered support surfaces by photofabrication techniques (see, e.g.^14^).

## The Simulation Model

We have chosen to model our hybrid thin films adapting the Gruhn-Hess-Luckhurst-Romano (GHLR) model^30–32^ which consists of a system of interacting centers (“spins”) placed at the sites of a regular lattice. The total Hamiltonian *U*_*N*_ is written as follows:

2 $${U}_{N}=\frac{1}{2}\sum _{\begin{array}{c}i,j\,\in \, {\mathcal F} ,\\ i\ne j\end{array}}{U}_{ij}+J\sum _{\begin{array}{c}i\,\in \, {\mathcal F} ,\\ j\,\in \,{\mathscr S}\end{array}}{U}_{ij},$$

where ${\mathcal F}$, ${\mathscr S}$ are the set of particles in the bulk and at the surfaces, respectively, and the parameter *J* corresponds to the strength of the coupling of the sample spins with the surface boundary spins (here we assume *J* = 0.5). The particles interact through the second rank nearest neighbour attractive pair pseudo-potential^32^:

3 $$\begin{array}{rcl}{U}_{ij} & = & {\varepsilon }_{ij}\{\lambda [{P}_{2}({{\bf{u}}}_{i}\cdot {{\bf{s}}}_{ij})+{P}_{2}({{\bf{u}}}_{j}\cdot {{\bf{s}}}_{ij})]+\mu [({{\bf{u}}}_{i}\cdot {{\bf{s}}}_{ij})({{\bf{u}}}_{j}\cdot {{\bf{s}}}_{ij})({{\bf{u}}}_{i}\cdot {{\bf{u}}}_{j})-\frac{1}{9}]\\  &  & +\,{P}_{2}({{\bf{u}}}_{i}\cdot {{\bf{u}}}_{j})\{\nu +\rho [{P}_{2}({{\bf{u}}}_{i}\cdot {{\bf{s}}}_{ij})+{P}_{2}({{\bf{u}}}_{j}\cdot {{\bf{s}}}_{ij})]\}\},\end{array}$$

where *ε*_*ij*_ = *ε*, *ε* > 0 for *i*, *j* nearest neighbours and 0 otherwise, **s**_*ij*_ = **r**_*ij*_/|**r**_*ij*_|, **r**_*ij*_ = **r**_*i*_ − **r**_*j*_, with **r**_*i*_, **r**_*j*_ the position vectors of the *i*-th and *j*-th lattice points; **u**_*j*_, **u**_*k*_ are unit vectors along the axis of the two particles (or “spins”) and *P*_2_ is a second rank Legendre polynomial. The parameters *λ*, *μ*, *ν*, *ρ* are defined in terms of the splay, twist and bend elastic constants *K*_11_, *K*_22_, *K*_33_ as

4 $$\lambda =\frac{1}{3}\Lambda (2{K}_{11}-3{K}_{22}+{K}_{33});$$

5 $$\mu =3\Lambda ({K}_{22}-{K}_{11});$$

6 $$\nu =\frac{1}{3}\Lambda ({K}_{11}-3{K}_{22}-{K}_{33});$$

7 $$\rho =\frac{1}{3}\Lambda ({K}_{11}-{K}_{33}),$$

with Λ a factor with dimensions of length. The spins represent a cluster of neighboring molecules whose short range order is assumed to be maintained through the temperature range examined^33^. We notice that the pseudo-potential in Eq. 3 is a mesoscopic, rather than a truly molecular one, as shown by the fact that it contains elastic constants, and thus it is temperature dependent. The one constant approximation case, i.e. *λ* = *μ* = *ρ* = 0, *ν* = −1, formally reduces Eq. 3 to the well-known Lebwohl-Lasher (LL) potential which correctly reproduces the orientational order characteristics of a nematic- isotropic (NI) phase transition^33^. This transition occurs, for the bulk LL, at a reduced temperature^27^ T^*^ ≡ *kT*/*ε* = 1.1232. While in simulating bulk systems^33^ periodic boundary conditions are employed in all three directions, in the present case we have a finite thickness film and boundaries in the vertical direction are implemented, as mentioned before, by considering additional layers of particles, kept fixed during the simulation, with suitable orientations chosen to mimic the desired surface alignment^33^. Here we consider uniaxial nematic films with hybrid conditions, homeotropic at the top and various degrees of ordering at the bottom, from a random planar to a perfect alignment along the *x* direction. The starting configurations of the lattice were chosen to be completely aligned along the *z* direction and the evolution of the system was followed according to the classic Metropolis Monte Carlo procedure^34^. The film was considered to be placed between crossed polarizers and polarizing microscope (POM) textures were simulated by means of a Müller matrix approach^35–37^, assuming that the molecular domains represented by the spins act as birefringent retarders on the light propagating linearly across the film. In practice the following parameters were employed for computing the optical textures: film thickness *d* = 5.3 *μ*m, ordinary and extraordinary refractive indices *n*_*o*_ = 1.5 and *n*_*e*_ = 1.66, and light wavelength *λ*_0_ = 545 nm.

## Simulation Results

**Figure 1:**
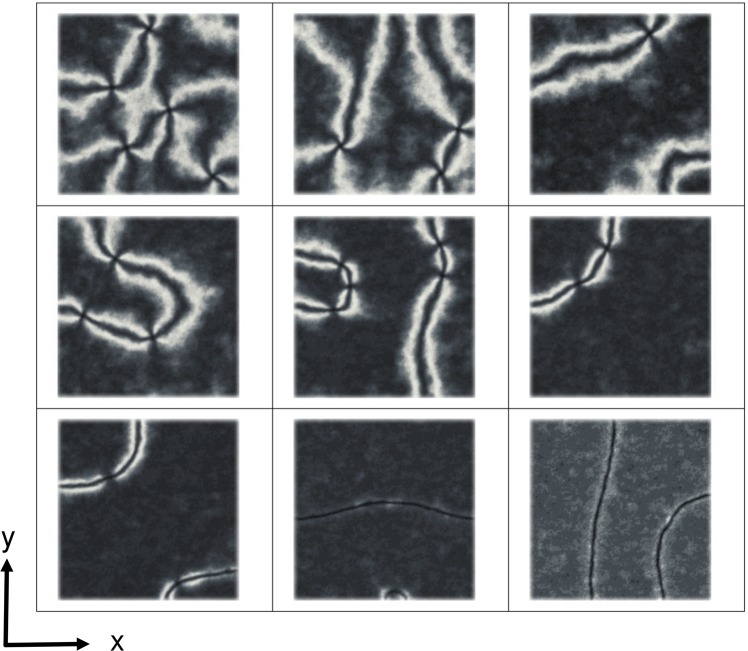
Optical images between crossed polars as obtained from a simulation of a 200 × 200 × (10 + 2) GHLR system with homeotropic alignment at *z* = *h* and different surface ordering at the bottom (*z* = 0). The frames (from top left to bottom right) refer to a surface order parameter 〈*T*_2_〉 = 0.00 (planar degenerate), 0.02, 0.05, 0.07, 0.10, 0.20, 0.30, 0.50 and 1.00 (perfect alignment along *x*). All have surface coupling *J* = 0.5 and elastic constants *K*_11_ = 7.0 pN, *K*_2_ = 4.4 pN, *K*_3_ = 9.7 pN.

We have considered a thin film of dimensions 200 × 200 × (*h* + 2), *h* = 10, system with the top and bottom boundary conditions implemented as described above. Empty (free) boundary conditions were employed instead at the other four lateral faces of the system. As we have shown in previous work^24^ the value of *J*, the strength of coupling with the surface spins, can be related to the film thickness, in the sense that increasing the strength of the anchoring is somehow equivalent to simulating thinner films. In the present case we have chosen a coupling corresponding to (1/2) of the molecular interaction strength, i.e. *J* = 0.5, value which is suitable to produce stable point defects for the hybrid nematic film with planar random orientation at the bottom. We have then considered various cases with increasing degree of surface ordering, 〈*T*_2_〉 at the bottom surface and the results are reported in Fig. 1. We see that, as 〈*T*_2_〉 increases, the point defects tend to lie on a line and that they then disappear forming a disclination line when the bottom surface spins are sufficiently aligned along *x*, with a (rough) critical threshold 〈*T*_2_〉 = 0.30–0.40 for the present case, where we have used the elastic constants of 5CB, i.e. *K*_11_ = 7.0 pN, *K*_2_ = 4.4 pN, *K*_3_ = 9.7 pN.

**Figure 2:**
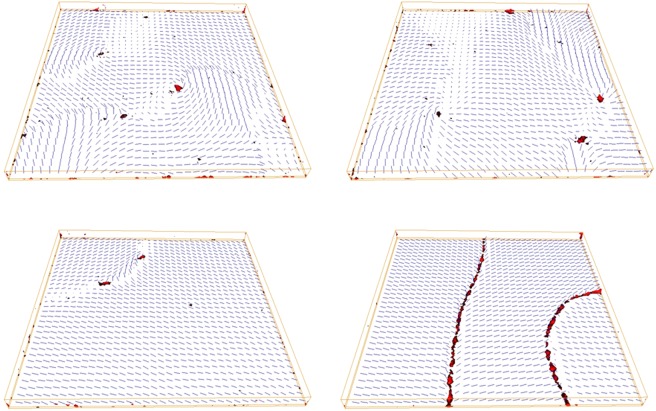
Plots of the local director at lattice sites and of the *c*_*l*_ Westin^38^ metric isosurfaces corresponding to a defect (red) of the simulated sample for some of the cases presented in Fig. 1 with different values of ordering at the bottom surface (*z* = 0). The frames refer to the first overlayer for a substrate with 〈*T*_2_〉 = 0.00, i.e. planar random (top-left), 2% (top-right), 20% (bottom-left), and 100%, i.e. perfect alignment along *x* (bottom-right). The axes are the same as in Fig. 1.

**Figure 3:**
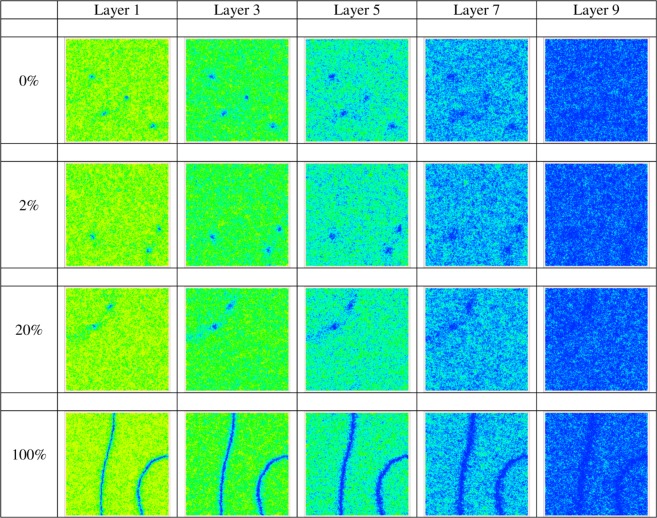
Snapshots of five layers of the nematic films starting from the bottom for four different percentages of ordering at the bottom surface of the film. Director field of the simulated samples for some values of ordering at the bottom surface corresponding to the cases presented in Fig. 2. The color of the spins varies from blue when they are oriented along z to yellow when they lie in the xy plane.

To visualize nematic director field and defects, as shown in Fig. 2, we have followed Callan- Jones *et al*.^3,38^, calculating the average ordering matrix U_*i*_ = 〈**u**_*i*_ ⊗ **u**_*i*_〉 at each lattice site *i*. The local director corresponds to the direction of the largest eigenvalue ${\lambda }_{1}^{i}$, while defects are identified from the so called Westin metric ${c}_{l}^{i}$, obtained taking the difference of the two largest eigenvalues: ${c}_{l}^{i}={\lambda }_{1}^{i}-{\lambda }_{2}^{i}$ and noting that ${c}_{l}^{i}$ tends to 1 for complete uniaxial order while it will tend to zero (or in practice have values below a given low threshold) at defects. We see that defects imprinted in the underlying partially disordered surface of the support are transmitted to the overlayer because of the rather strong surface anchoring imposed with the coupling parameter *J*. The effect of the ordering along *x* at the bottom surface is evident when looking at the cases shown in Fig. 2. In Fig. 2 (top-left), which corresponds to a planar degenerate surface, two +1 and two −1 defects are visible, while in the other extreme of homogeneous in plane order Fig. 2 (bottom-right) the lack of imprinted orientations covering all the angular directions in the *xy* plane does not allow +1 and −1 defects and we see instead a line of $+\frac{1}{2}$ and of $-\frac{1}{2}$ defects in the family of *xz* plane cutting through the film. It is also apparent that the defects start at the lowest layers of the system and link the spin orientations induced by the bottom surface to the perfectly homeotropic alignment at the top (Fig. 3).

**Figure 4:**
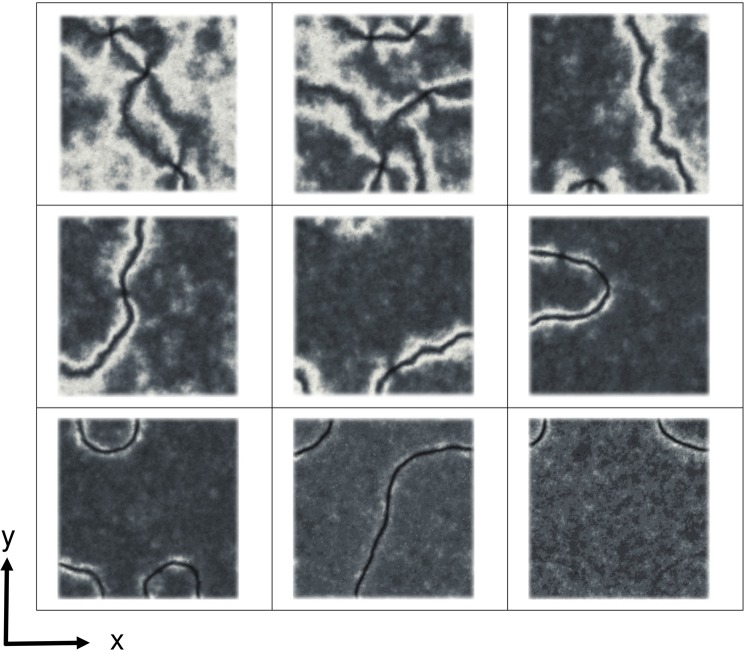
Polarized optical images as obtained from a simulation of a 200 × 200 × (10 + 2) Lebwohl-Lasher system where the three elastic constants are equal. The geometry of the system is the same as for the Gruhn-Hess case and the ordering at the bottom surface, 〈*T*_2_〉, is (from top left to bottom right): 0 (planar random), 0.02, 0.05, 0.07, 0.10, 0.20, 0.30, 0.50 and 1.00 (perfect alignment along *x*)). The coupling with the surfaces was choosen to be *J* = 0.5.

We notice that this scenario is due mainly to the specific type of boundary conditions and do not appear to depend on the different values of the elastic constants as can be verified by performing a simulation of a Lebwohl-Lasher model (which corresponds to the one elastic constant approximation) with the same surface order parameters (see Fig. 4). The effect of the difference in the elastic constants is to enhance the creation and stability of the point defects that continue to be present up to a large value of ordering at the bottom surface (see Fig. 1).

## Experimental Approach

Optical experiments were performed to verify the behavior seen in simulations. A 6 *μ*m hybrid liquid crystal cell was prepared using a substrate with uniform homeotropic anchoring and one with planar anchoring with increasing degrees of uniaxial order. The substrate with homeotropic anchoring was prepared by spin coating a homeotropic aligning layer (JSR, JALS-2021-R2) on a glass substrate. The substrate with planar anchoring was prepared by spin-coating a photoaligning layer (DIC, LIA-03), and subsequently performing maskless photoalignment to create surfaces with orientational easy axis distributions with varying uniaxial order. Maskless photoalignment was performed using a home-built system comprising a LC display (LCD) projector, projection optics, and a rotating waveplate, PC-controlled, to sequentially project linearly polarized light at 1° increments (corresponding to 180 phase-levels) at predefined positions^39^. The pixel resolution of the LCD projector was 1024 × 768 with a pixel size of approximately 1.5 × 1.5 *μ*m^2^. The photoaligning layer was azobenzene-based, and chosen so as to set the orientational easy axis parallel to the substrate, in a direction perpendicular to the incident polarization. The wavelength of the patterning light was 436 nm.

**Figure 5:**
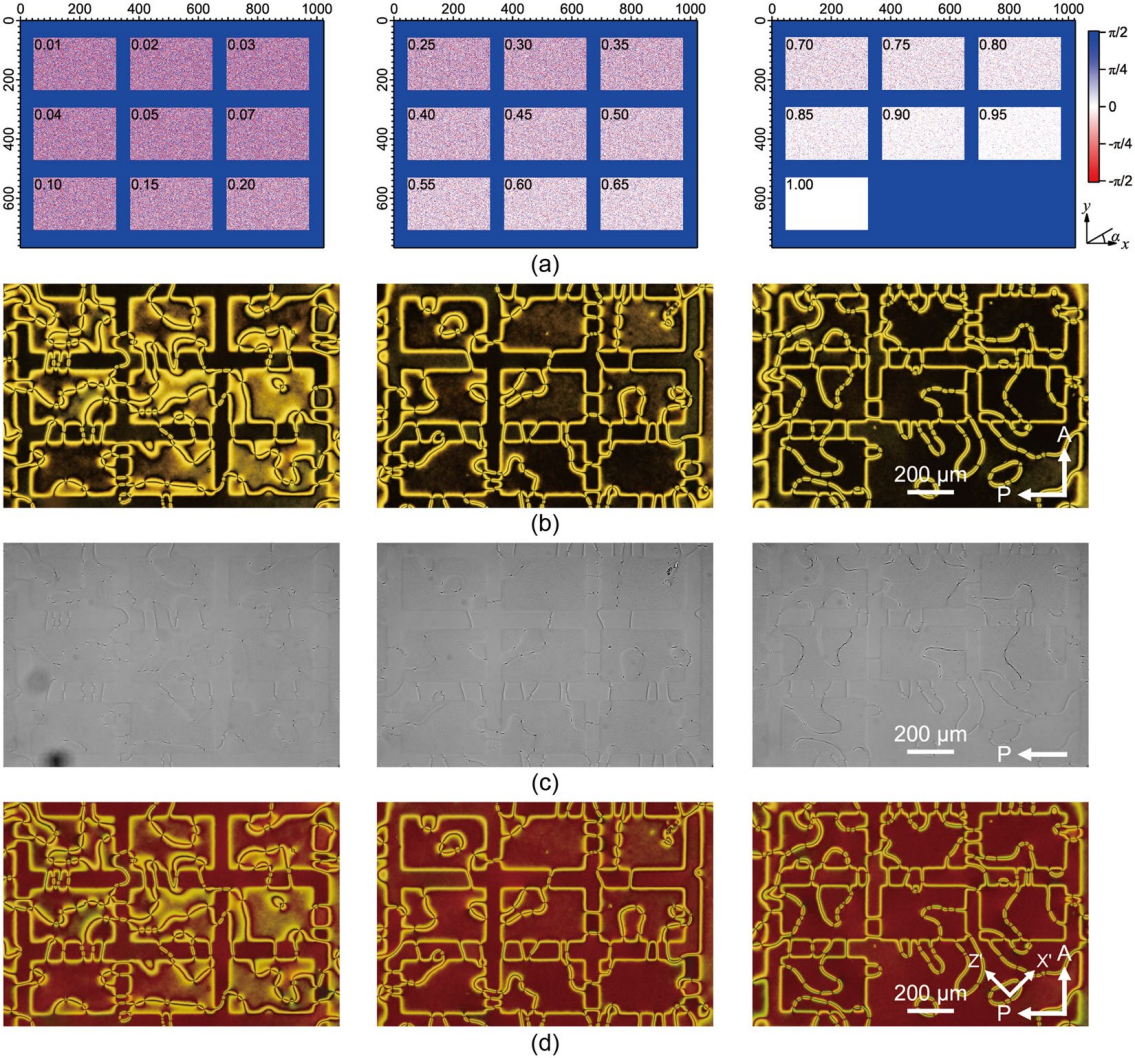
(**a**) Design of the easy axis distribution created in experiment, where the values written in each of the 25 regions indicate the designated 〈*T*_2_〉 values. (**b**) Experimental POM images of the prepared hybrid cell. (**c**) Optical Microscopy images with the analyzer removed. Images have been converted to greyscale and the brightness and contrast were adjusted using Adobe Photoshop (using the same parameters for the three images) to enhance the difference in the defect textures. (**d**) POM images with a first order retardation plate (Δ = 530 nm).

Within one projection area, 9 regions with 278 × 178 pixels were prepared, each possessing different degrees of uniaxial order along the horizontal (*x*) direction. Three different sets of projections were made to create 25 regions covering 〈*T*_2_〉 values of 0.01 to 1.00. To realize a degree of bottom surface ordering corresponding, as far as possible, to that of the MC simulations, we have first randomly chosen the position of the desired percentage of pixels (or “spins” of the lattice model) where an alignment along *x* is then photoimprinted, and successively we have extracted from a uniform distribution the orientations at the remaining pixels so as to complete the pattern in each of the 25 regions. Between the various regions, a gap with uniaxial orientation along the *y* direction was set. We notice that this arrangement, albeit similar, is not identical to the computer simulated one, where each region was treated independently from the others, thus avoiding by construction the possibility of cross-talk between the regions. The photoalignment pattern is shown in Fig. 5(a), where the degree of uniaxial order along the *x* horizontal direction increases from top-left to bottom right in each projected area. The region outside of the overall projection area was left untreated.

After cell preparation, a photopolymerizable nematic LC mixture, (RMM141C, Merck) was injected in the gap for observation of the optical texture by (POM, Nikon, LV100N POL). The reactive mesogen RMM141C was chosen because this LC showed a clear Schlieren texture on the photoalignment layer used, whereas other nematic LCs, for example the well-known 4-pentyl-4′ cyanobiphenyl, showed the marble texture^40^. The sample was heated to 70 °C, which is above the clearing point of the nematic (*T*_*iso*_ ~ 67 °C), and gradually cooled to 42 °C on a commercial hotplate (Instec, HCS622VXY-F4SYS). Because of the limited sample size, the defects tended to escape out of the patterned region after several 10 minutes; by photopolymerizing the texture after confirming that different textures appeared in the patterned regions.

## Results and Discussions

**Figure 6:**
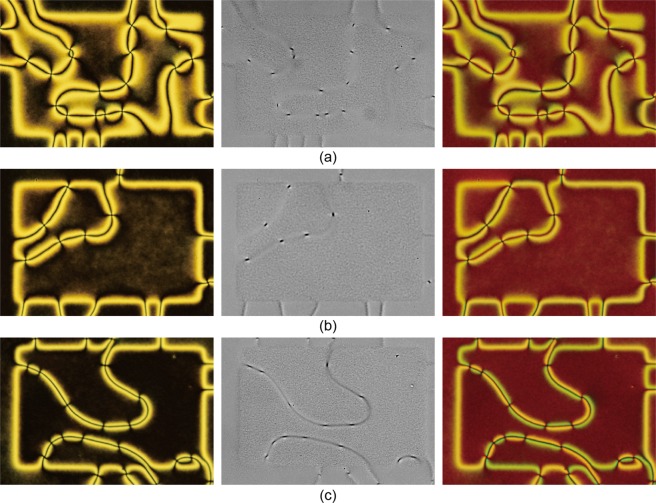
Magnified images of regions with (**a**) 〈*T*_2_〉 = 0.01, (**b**) 0.45, and (**c**) 1.0. The orientations of the polarizers and the waveplate are the same as Fig. 5.

Figure 5(b) shows POM images of the sample observed between crossed polarizers. One can recognize the 25 photoaligned regions, showing different optical textures depending on the value of 〈*T*_2_〉. In the regions with low uniaxial order, a schlieren texture is observed, which cannot be distinguished from that observed in the region without the patterning. However, as the uniaxial surface order is increased, the region gradually becomes darker, and shows string-like textures. The difference is apparent also when the analyzer is removed (Fig. 5(c)); in regions with low uniaxial order, isolated point defects are visible, whereas in high-order regions, an apparent line defect is observed. Figure 6 shows magnified images of regions with 〈*T*_2_〉 = 0.01, 0.45, and 1.0 to enhance the change in the optical textures as the bottom ordering increases.

The first general observation is that there is at least a qualitative agreement between simulations and real experiments. In simulations (for the LL model, Fig. 4, corresponding to the one elastic onstant approximation), a transition occurs from the standard schlieren texture (dark brushes in a bright background) to a more line-like texture at approximately 〈*T*_2_〉 ~ 0.07–0.1, where two of the four dark brushes emanating from a point defect become elongated and surrounded by two narrow bright bands. This transition is observed at 〈*T*_2_〉 ~ 0.1 in experiment, which is a satisfactory agreement considering that the simulations deal with an idealized liquid crystal and no attempt has been made to fit the parameters of the actual material used in experiments. For the case of different elastic constants the transition appears to move to different values of 〈*T*_2_〉, see for example Fig. 1, but the main features of the behaviour do not change.

**Figure 7:**
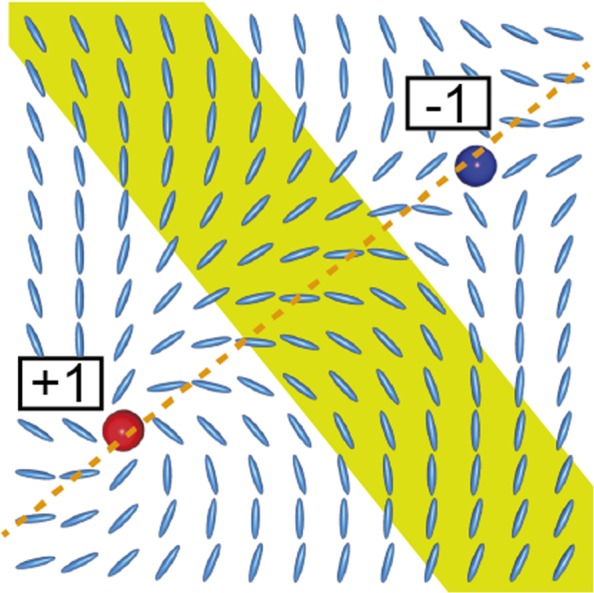
Topological requirement of *π* director rotation (yellow stripe) between two oppositely charged point defects.

With an increase in surface order, the width of the bright bands becomes narrower, in both simulations and experiments. However, simulations show a near-complete suppression of light leakage at 〈*T*_2_〉 > 0.5, whereas in experiment the bright bands remain. We attribute this difference to the fact that point defects were observed in experiment in all regions including 〈*T*_2_〉 = 1.0, whereas simulations gave none in this limit. Detection of the defect strength by rotating the sample indicated that defects have strengths of either + or −1, and the optical texture (both schlieren and line-like) occurs as to connect oppositely charged defects. Figure 5(d) shows a POM image of the sample taken after inserting a first order retardation plate (Δ = 530 nm) in the optical path, demonstrating the alternation in the azimuthal tilt direction in each segment of the line-like texture. Point defects impose topological boundary conditions such that the director must rotate in a specific direction around the defect core, and in addition, since oppositely charged defects are connected, a *π* rotation in the director must occur along the normal of a line that connects two point defects (Fig. 7). Since this topological constraint requires some regions to be aligned in directions not parallel to the polarizers, light leakage must occur.

It is very difficult to compare the anchoring strength which results from the complex fabrication process described in experiment and that employed in the simulations, which is probably significantly higher than that in experiment. If this is the case, as we believe, this factor strongly disfavors the deformations associated with point defects in the simulated textures. Also, since the nematic phase is experimentally obtained by cooling from the random isotropic phase, the point defects formed at the phase transition may have entered a metastable state and remained as a singularity. Furthermore we have boundary conditions in the *x* − *y* plane, i.e. at the four sides of the sample cell, that are “empty” in the case of the MC simulations, while in the real samples we have narrow oriented frames that are part of an overall, somehow connected surface area, as shown by the continuity of the patterns between neighbouring cells in Fig. 5(b). Given all these possible sources of discrepancy it is comforting to observe that the textures and their change upon increasing the surface order are quite similar, and we take this as a validation of the computational results. This being the case, we can then use the ability of simulations to examine the director field and its defects across the film thickness, adding a visualization capability in three dimensions that complements the *xy* projections observed from POM. For instance, we see from Fig. 2 at a 2% planar order (top right) that the line feature actually connects opposite sign point defects, as found in experiment. Simulations also indicate that the defect region is in all cases adjacent to the bottom partially ordered surface and that the defects appear as vertical escaped walls rather than as disclination lines, as they appear from the inevitably two dimensional projection offered by POM. We notice, however, that the fact that the defects originate close to the planar anchoring surface appears to be at variance with respect to the results of ref. ^28^, where disclinations lines are observed in a hybrid cell but the disclination lines appear to be located in the middle of the sample. On the other hand this could be due to pinning at the silica microspheres used as spacers, as stated in^28^, which are not present in our case neither in the simulations nor, as mentioned before, in the experiment. We believe that the curvature of the spheres, necessarily non-negligible across the sample thickness and the anchoring of orientation at their silica surface, which is not specified and should depend on their pre-treatment could provide significantly different boundary conditions.

## Conclusions

In the present study we have investigated, by a combination of Monte Carlo simulations and real experiments, a hybrid cell where one (the top one in our case) of the two facing surfaces is always inducing homeotropic alignment, while the other has an in plane ordering varying from fully random to perfectly homogeneous. We have found, that a random, degenerate, anchoring favors point defects, while homogeneous alignment favors the appearance of filament like defects. Tuning the in plane order of the bottom surface to values intermediate between the two extremes allows to follow the change over from one type of defect to the other. In particular, performing simulations with anisotropic and isotropic elastic constants, we have observed that the transition is mainly due to the surface alignment rather than on the details of the nematic elastic constants. Examining snapshots of the film layer by layer (Fig. 3) it appears that the transitions from planar to vertical alignment is a sudden one, starting from the bottom overlayer, rather than from an intermediate one. We believe that this finding is an example of a major innovation of the present work: the combined use of Monte Carlo simulations and optical experiments. On one hand the 2D polarized microscopy textures obtained from experiment can validate the MC simulations. On the other, the simulations, once validated, allow a detailed visualization of the 3D molecular organization across the film, complementing the 2D projections produced by POM experiments and offering an important way of discriminating between models.
